# Association between Active *Helicobacter pylori* Infection and Glaucoma: A Systematic Review and Meta-Analysis

**DOI:** 10.3390/microorganisms8060894

**Published:** 2020-06-13

**Authors:** Michael Doulberis, Apostolis Papaefthymiou, Stergios A. Polyzos, Panagiotis Bargiotas, Christos Liatsos, David Shiva Srivastava, Christos Zavos, Panagiotis Katsinelos, Jannis Kountouras

**Affiliations:** 1 Division of Gastroenterology and Hepatology, Medical University Department, Kantonsspital Aarau, 5001 Aarau, Switzerland; 2 Department of Internal Medicine, Second Medical Clinic, Ippokration Hospital, Aristotle University of Thessaloniki, 54642 Thessaloniki, Macedonia, Greece; appapaef@hotmail.com (A.P.); czavos@ymail.com (C.Z.); akis_katsinelos@yahoo.gr (P.K.); 3 First Laboratory of Pharmacology, School of Medicine, Aristotle University of Thessaloniki, 55134 Thessaloniki, Macedonia, Greece; spolyzos@auth.gr; 4 Department of Emergency Medicine, University Hospital Inselspital Bern, Bern, 3010 Bern, Switzerland; davidshiva.srivastava@insel.ch; 5 Department of Gastroenterology, University Hospital of Larissa, 41110 Larissa, Greece; 6 Department of Neurology, Medical School, University of Cyprus, Nicosia 2029, Cyprus; bargiotas.panagiotis@ucy.ac.cy; 7 Department of Gastroenterology, 401 General Military Hospital of Athens, 11525 Athens, Greece; cliatsos@yahoo.com

**Keywords:** *Helicobacter pylori* infection, glaucoma, primary open-angle glaucoma, pseudo-exfoliation glaucoma

## Abstract

Background: Glaucoma is the second most common cause of blindness worldwide affecting almost 70 million individuals. *Helicobacter pylori (H. pylori)* is a widespread pathogen with systematic pathogenicity. This meta-analysis aimed to estimate the contradictory data regarding a potential association between active *H. pylori* infection and glaucoma. Materials and Methods: A research in MEDLINE/PubMed and Google Scholar was conducted and original studies investigating the relationship between *H. pylori* infection and glaucoma were included. Analysis was performed with random effects model. The main outcome was the odds ratio (OR) with 95% confidence intervals (CI) of *H. pylori* infection as a risk factor for glaucoma. A parallel analysis studied the role of active infection as indicated by histology and the titer of anti-*H. pylori* antibodies. For the anti-*H. pylori* antibody titers, weighted mean differences (WMD) were estimated between patients and controls. Results: Fifteen studies were included, with 2664 participants (872 patients with glaucoma and 1792 controls), divided into primary open-angle glaucoma (POAG), normal tension glaucoma (NTG) and pseudo-exfoliation glaucoma (PEG). The association between *H. pylori* infection and overall glaucoma was significant (OR = 2.08, CI 95% 1.48–2.93) with moderate heterogeneity (*I*^2^ = 61.54%). After stratification by glaucoma subtype, heterogeneity was eliminated in the NTG subgroup. Studies with healthy controls, and controls with anemia yielded very low or no heterogeneity, respectively. Gastric biopsy to document active *H. pylori* infection yielded the highest OR (5.4, CI: 3.17–9.2, *p* < 0.001) and null heterogeneity. For anti-*H. pylori* antibody titers, there was a significant difference in WMD between patients and controls (WMD 15.98 IU/mL; 95% CI: 4.09–27.87; *p* = 0.008); values were greater in glaucoma patients, with high heterogeneity (*I*^2^: 93.8%). Meta-regression analysis showed that mean age had a significant impact on glaucoma (*p* = 0.037). Conclusions: Active *H. pylori* infection may be associated with glaucoma with null heterogeneity, as, beyond histology, quantified by anti-*H. pylori* titers and increases with age.

## 1. Introduction

*Helicobacter pylori* (*H. pylori*) is a Gram-negative spiral-shaped bacterium [1] with worldwide distribution. About 58% (varying from 39.9–91.7%) of the global population is estimated to be colonized with *H. pylori* and its prevalence is even higher in less-industrialized countries [2,3,4]. *H. pylori* can cause chronic gastritis, peptic ulcers, gastric adenocarcinoma, as well as mucosa-associated lymphoid tissue lymphoma [5,6,7,8,9,10]. Beyond this local pathogenicity, there appears to be a variety of *H. pylori*-associated extragastric manifestations [11,12,13,14], including Alzheimer’s disease (AD), primary open-angle glaucoma (POAG), also called “ocular AD” and pseudoexfoliative glaucoma (PEG) at least in certain populations. Glaucoma, leads to the death of retinal ganglion cells and their axons and is the second most frequent cause of blindness worldwide. It affects 66.8 million individuals, causes bilateral blindness in 6.7 million people [15,16,17], and is a neurodegenerative disorder of the optic nerve, with the hallmark of increasing “cupping” of the optic disc [18].

Studies evaluating the potential relationship between *H. pylori* infection and POAG have reported either positive or no association, so that there is still controversy. A conceivable explanation of this discrepancy is that the serological test used by several studies does not discriminate between active and past infections [19,20,21,22,23,24]. It is important to note that only active *H. pylori* infection induces humoral and cellular immune responses that, due to molecular mimicry, cross-react with components of host nerves, thereby inducing apoptotic injury to extragastric tissues and may contribute to the pathophysiology of certain pathologies such as Guillain–Barré syndrome [25,26,27] and autoimmune pancreatitis [28,29,30] and possibly in glaucoma **[6,31,32]** or other neurodegenerative disorders [33,34,35]. As a potential exception, high anti-*H. pylori* Immunoglobulin (Ig)G titers appear to be associated with the degree of gastritis and mucosal bacterial concentration [36]; anti-*H. pylori* titer is associated with the gastric bacterial burden; and major reductions in *H. pylori* infiltration and serum anti-*H. pylori* titers are recorded after *H. pylori* eradication [37]. Thus, the serum high anti*-H. pylori* titer might be an index of *H. pylori* load in patients with active infection.

We were motivated by these controversies to conduct a systematic review and meta-analysis of observational studies, in order to evaluate the association between glaucoma and active *H. pylori* infection documented by gastric biopsy. We also investigated whether the titer of serum anti-*H. pylori* IgG antibodies was associated with glaucoma.

## 2. Methods

### 2.1. Strategy of Bibliographic Search

**Figure 1:**
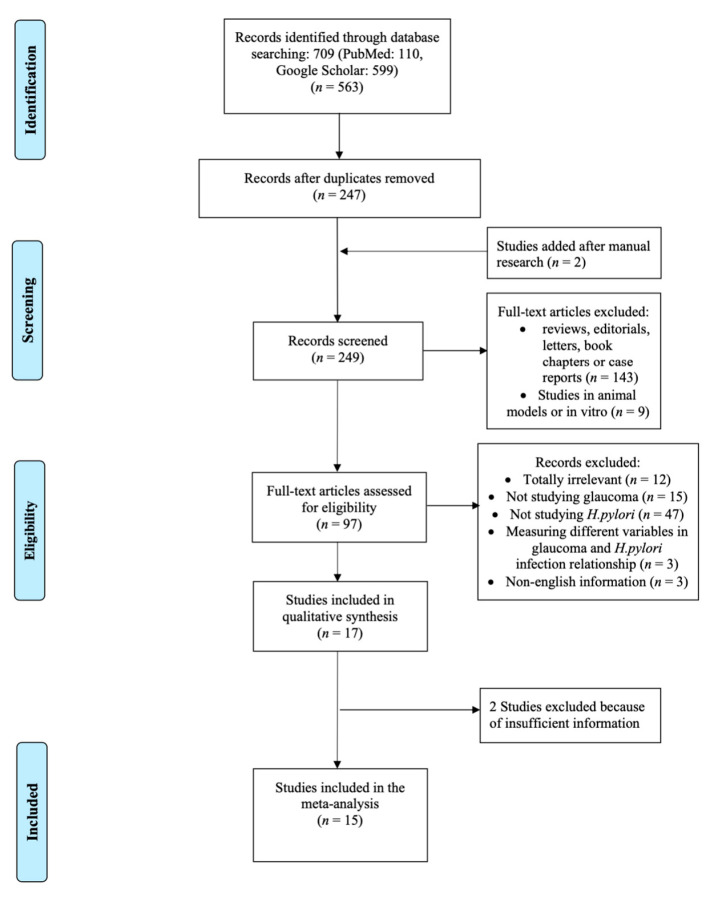
Flowchart based on Preferred Reporting Items for Systematic Reviews and Meta-Analyses (PRISMA) guidelines interpreting the selection process of the reviewed studies to insert in the meta-analysis.

The study protocol was in accordance with the reporting guidelines for the Meta-analysis of Observational Studies in Epidemiology (MOOSE) [38]. The search strategy and flowchart (Figure 1) were performed according to the Preferred Reporting Items for Systematic Reviews and Meta-Analyses (PRISMA) [39]. An initial online search was confined to English language literature from 1 January 1960 to 30 September 2019. The search queries included the following Boolean search terms, modified according to the demands of each database; (“*Helicobacter pylori*” OR “*H. pylori*” OR *Hp* OR “*Campylobacter pylori*”) AND (glaucoma OR “open-angle glaucoma” OR “primary open-angle glaucoma” OR POAG OR “ocular hypertension” OR “pseudo-exfoliation glaucoma”). We searched the MEDLINE/PubMed and Google Scholar databases. Furthermore, additional relevant articles were searched in the reference lists of the retrieved articles, as well as by using the “similar article” function of PubMed. Unpublished works, abstracts, and oral or poster presentations were excluded. If we required additional data, e-mails were sent to the first and/or the corresponding authors. A database of all retrieved articles was created and managed with the reference manager Mendeley Desktop for Windows v. 1.19.1 (Mendeley Ltd., Elsevier, Amsterdam, The Netherlands).

### 2.2. Inclusion–Exclusion Criteria

Original, full-text observational studies were included (cross-sectional, case-control or cohort studies).

Studies were excluded if: the diagnosis of glaucoma was not clearly stated; the diagnosis of *H. pylori* infection was biased (e.g., patients recently treated with antibiotics or proton pump inhibitors); the data included incomplete and/or the corresponding author did not provide additional records; the publications did not contain original data, (e.g., letters to the editor, editorials, commentaries, reviews, meta-analyses); or if they evaluated fewer than seven points in the quality assessment process using the Newcastle–Ottawa scale (NOS).

The control groups of all studies were assessed and the least-acceptable conditions were the absence of glaucoma-related ocular diseases, or *H. pylori*-related gastric diseases (dyspepsia, peptic ulcer, gastric cancer, mucosa-associated lymphoid tissue lymphoma) and major co-morbidities (such as cancer, liver cirrhosis and cardiovascular or renal failure).

### 2.3. Data Extraction and Assessment for Study Quality

Two investigators (M.D. and A.P.) independently first reviewed the title and abstract of the retrieved studies. After this initial screening, they evaluated the content of the full text of the remaining articles and their quality. If a disagreement emerged, this was resolved by the senior investigator (J.K.) and then a joint review was performed, in order to reach consensus. If there was more than one article on a single study (implying potential patient overlap), the most recent publication was included in the meta-analysis.

Information was then extracted into the electronic database, as follows: (1) first author’s name, type of study, journal and year of publication; (2) country of origin and race, patient and control numbers and their mean age; (3) method of *H. pylori* infection diagnosis; (4) final outcomes expressed in odds ratio (OR). An individual file was created for studies that calculated the anti-*H. pylori* titers and all medians and standard deviations (SD) were additionally extracted for both groups.

### 2.4. Quality Assessment

The quality of the included studies was assessed by using the Newcastle–Ottawa scale (Ottawa Hospital Research Institute, Ottawa, ON, Canada) [40]. In cases of conflicting ratings between the two reviewers (M.D. and A.P.), the arbitration of J.K. was required.

### 2.5. Outcomes

The association between *H. pylori* infection and glaucoma patients and controls was expressed by OR (95% confidence intervals (CI)). To assess potential heterogeneity, subgroup analysis and meta-regression analysis were conducted. Weighted mean differences (WMD) were used to evaluate differences in anti-*H. pylori* antibody titers between patients and controls.

### 2.6. Statistical Analysis

All statistical analyses were performed with STATA v.14.0 software for OSX (Stata Corp., College Station, TX, USA). The fundamental inserted commands included “metan”, “metareg”, “metafunnel” and “metabias”.

As heterogeneity among the studies was suspected, a random effect model was implemented. The heterogeneity among the analyzed studies was evaluated by three tools; the *τ*^2^ test, the *χ*^2^ test and *I*^2^. If the *Q* value exceeded the degrees of freedom with *p* < 0.05, heterogeneity was consistent [41]. Subgroup analyses were performed to investigate potential factors causing heterogeneity by evaluating the effect of independent categorical variables (i.e., race, glaucoma subtype, *H. pylori* infection diagnostic method). Likewise, meta-regression was performed to estimate the effect of continuous variables (i.e., mean age, male to female ratio, anti-*H. pylori* antibodies level). For the variable “race”, studies originating in Europe and America were included in the “West” group, whereas studies from Asia were in the “East” group and those from Africa in a distinct group.

Anti-*H. pylori* antibody titers in patients and controls were compared in studies that provided sufficient quantitative information about anti-*H. pylori* antibody titers that could be inserted into our model [22,24,34,42]. Subgroup and meta-regression analyses were applied as previously described**.**

As the authors have a special interest in extra-gastric manifestations of *H. pylori* infection, this might be associated with specific bias. Therefore, a sensitivity analysis was performed by excluding all studies of our group. Any potential publication bias was examined with Begg’s and Egger’s tests, with *p* < 0.05 in both tests indicating publication bias.

## 3. Results

### 3.1. Literature Search and Characteristics of the Studies

The flowchart according to the PRISMA guidelines is depicted in Figure 1. The initial search yielded a total of 709 articles. After removal of duplicates (462), 247 articles remained for the further screening process, which included checking titles and abstracts, followed by reviewing the full text manuscript of the eligible studies (*n* = 97). Eventually 17 studies were included in the systematic review. Two of these were excluded, as extra data were needed and these were not provided by the corresponding authors, and/or the methodology was doubtful [43,44]. Therefore, 15 final studies were considered for the meta-analysis and included 2664 participants (872 patients with glaucoma and 1792 controls) with three types of glaucoma (POAG, NTG, PEG) [19,22,23,24,34,41,42,45,46,47,48,49,50,51,52]. One potential type (ocular hypertension), reported by Galloway et al. [24] was excluded because it did not meet our own inclusion criteria.

Study characteristics and the respective outcomes are shown in Table 1. Most of the studies were carried out in Asia (*n* = 8), followed by Europe (*n* = 5). In Africa and in America, one single study per continent was conducted. Almost half of the included articles were case-control (*n* = 7) and the rest cohort studies.

A final evaluation of the included studies was performed on the basis of the NOS, except for one study, which provided all the necessary information for our analysis in the English abstract, even though the main text was in Persian (Farsi) [50]. All of the evaluated studies met a score of more than seven (7).

The authors of all included studies considered their results separately for each glaucoma subtype and we followed this classification by creating 23 records derived from the 15 studies included. In order to investigate the effect of anti-*H. pylori* antibody titers on glaucoma, we extracted data from 8 datasets, which clearly provided mean values and standard deviations of this parameter.

### 3.2. Overall Results and Subgroup Analyses

**Figure 2:**
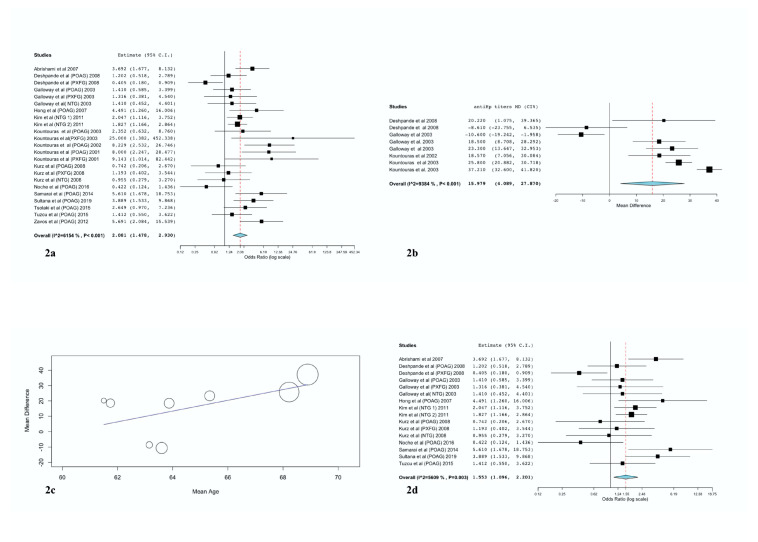
(**a**) Forest plot illustrating, in OR, the overall and per included study association between *H. pylori* infection and glaucoma risk. (**b**) Forest plot for the association between means of difference of anti- *H. pylori* antibodies titers and glaucoma occurrence. (**c**) Meta-regression scatter plot indicating the relationship between the means of difference of anti-*H. pylori* antibodies titers and glaucoma risk after adjustment for mean age. (**d**) Sensitivity analysis excluding our group’s studies and confirming the absence of bias because of our own studies. CI, confidence interval; *H. pylori*; *Helicobacter pylori*; NTG, normal tension glaucoma; OR, odds ratio; POAG, primary open angle glaucoma; PEG, pseudo-exfoliation glaucoma; MD, weighted mean differences.

The overall association between *H. pylori* infection and glaucoma was statistically significant (OR = 2.08, CI 95% 1.48–2.93) (Figure 2a). As the heterogeneity was moderately high (*I*^2^ = 61.54%) it was necessary to check for potential cofounders that might possibly have affected the results. We therefore also performed subgroup analysis (Table 2) and meta-regression analysis. After stratification by glaucoma subtype or region, the heterogeneity for each region remained essentially unchanged. There was only a statistically significant reduction in the heterogeneity of the NTG subgroup.

It was investigated whether the control group influenced the results. Studies with cataract controls did not lead to a significant association between *H. pylori* infection and glaucoma. In this subgroup, studies with healthy controls provided acceptable heterogeneity (*I*^2^ = 23.07%), whereas studies with anemia controls yielded null heterogeneity.

It was also investigated whether the results were influenced by the method of *H. pylori* infection detection. Gastric mucosa histology for *H. pylori* infection documentation [51] gave the highest OR of all methods (5.4, CI: 3.17–9.2, *p* < 0.001), but also null heterogeneity. By contrast, studies that used the urea breath test (UBT) did not show any significant association between *H. pylori* infection and glaucoma. Finally, meta-regression analyses did not reveal any benefit of continuously measured variables on the overall outcome and heterogeneity.

Comparison of the WMD of anti-*H. pylori* antibody titers between patients and controls gave a statistically significant difference (WMD 15.98 IU/mL); this was greater in glaucoma patients than in controls, albeit with high heterogeneity (*p* = 0.008, CI: 4.09–27.87, *I*^2^: 93.8%) (Figure 2b). Subgroup analysis could not explain the source of the heterogeneity. However, the meta-regression analysis proved that mean age had a statistically significant impact on the results (*p* = 0.037). More specifically, as Figure 2c illustrates, increasing age was associated with greater WMD of anti-*H. pylori* titers in glaucoma patients than in controls.

### 3.3. Sensitivity Analysis

We excluded our own studies, and then repeated the analysis (Figure 2d), including 16 datasets. The derived OR (1.55, CI 95%: 1.1–2.2) indicated a positive relationship between *H. pylori* infection and glaucoma, with similar heterogeneity to the initial analysis.

### 3.4. Publication Bias

**Figure 3:**
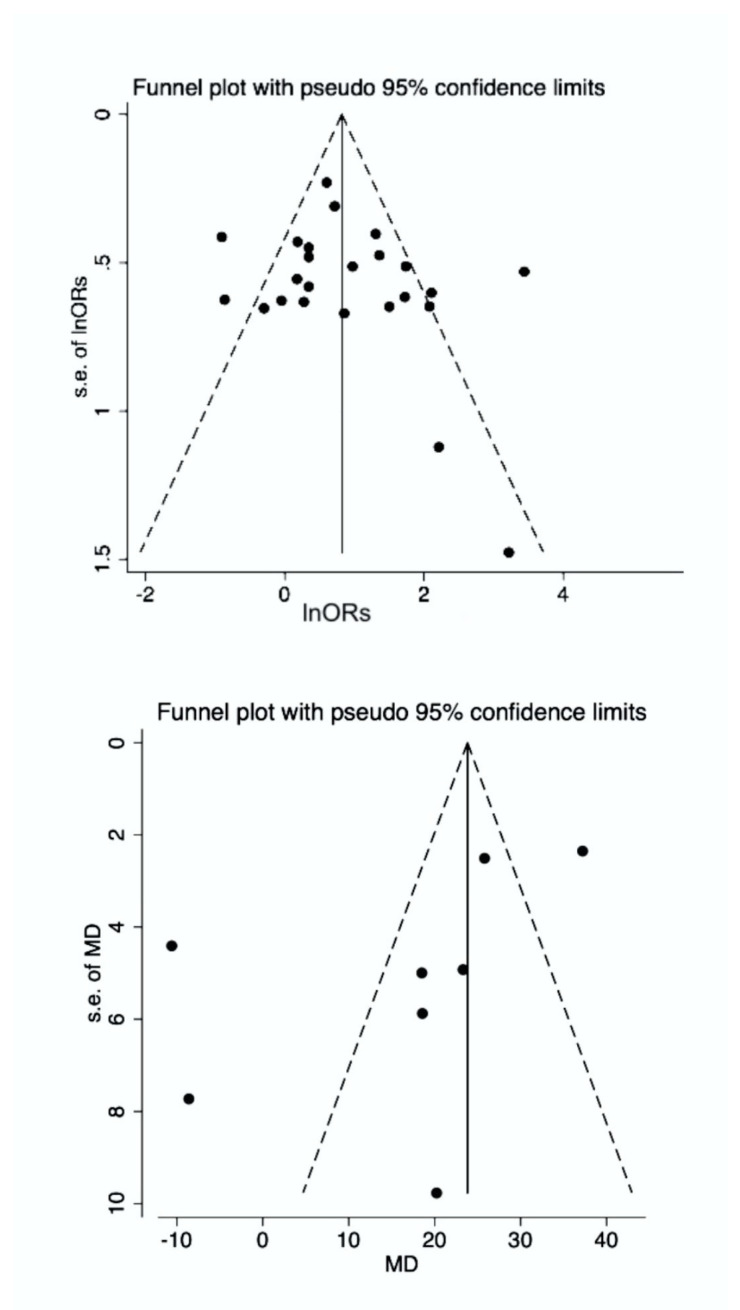
Funnel plot for both study models**.** Despite the non-significant Egger and Begg tests, there is a visual asymmetry in the funnel plots, indicative of a degree of publication bias. MD; weighted mean differences, OR; odds ratio, SE; standard error.

For the association between *H. pylori* infection and glaucoma, Egger and Begg tests provided *p* values of 0.347 and 0.174, respectively, thus eliminating the possibility of publication bias (Figure 3). Similarly, for the comparison of the WMD of anti-*H. pylori* antibody titers between patients and controls, the Egger and Begg tests provided *p* values 0.266 and 0.107. Nonetheless, despite the non-significant tests, there is a visual asymmetry in the funnel plots, which should be considered.

## 4. Discussion

Glaucoma is a major economic burden, with as many as 80 million patients by 2020 [17,53,54]. To our knowledge, this meta-analysis demonstrated, for the first time, a strong association between glaucoma and active *H. pylori* infection as documented by histology (Table 2) with total lack of heterogeneity. Relative data indicate that histology displays the higher sensitivity and specificity than the UBT and the rapid urease test (RUT) for the diagnosis of active *H. pylori* infection [55,56]. The cited sensitivity and specificity are 80–95% and 99–100%, respectively [55]. Studies in which *H. pylori* infection was diagnosed by UBT did not indicate a significant association, although this requires careful interpretation due to the small study number (*n* = 2). Furthermore, the selection of controls seems to affect this association. The association was less robust when cataract patients were used as controls, whereas healthy or anemic controls maintained the positive relationship and yielded low heterogeneity (Table 2). Thus, if histology is to be routinely used for *H. pylori* infection documentation, given its great correlation to the “gold-standard” *H. pylori* culture and its superiority to the other methods [55,57], further large-scale prospective studies are necessary to confirm this strong association with zero heterogeneity.

Moreover, a novelty of this meta-analysis is the quantification of the aforementioned association between *H. pylori* infection and glaucoma by using the anti-*H. pylori* IgG titer to reflect potentially active *H. pylori* infection; in clinical terms, the higher anti-*H. pylori* titers, the higher possibility of glaucoma—an association that is strengthened by aging. A possible hypothesis is that aging is simply a common denominator for both *H. pylori* infection and glaucoma. However, mechanistic studies may help to explain how aging mediates this association. Recently, the concept of triggering autoimmune disorders after bacterial infections through antigenic similarities was applied to *H. pylori* infection, thus unraveling the molecular mimicry between *H. pylori* antigens and host proteins-targets of autoantibodies in many conditions [58,59]. In this regard, the decline of the immune system due to aging is reflected in the augmented susceptibility to autoimmune disorders [60,61,62] and autoimmunity has been postulated to play an important role in glaucomatous injury to the optic nerve, [31,63,64] so that autoantibodies against proteins in retina and/or the optic nerve might be involved in glaucomatous neuropathy of the optic nerve [63,64]. Autoimmune injury to the optic nerve may occur directly by autoantibodies or indirectly by way of a mimicked autoimmune response to a sensitizing antigen, which in turn might damage retinal ganglion cells [63,64,65,66]. Autoantibodies against proteins in the retina and/or the optic nerve, resulting from the possible molecular mimicry between *H. pylori* and ocular proteins, might be involved in glaucomatous neuropathy of the optic nerve [63,64]. More specifically, *H. pylori* antigens stimulate the production of specific antibodies which recognize homologous host protein sequences, such as in glycoproteins, heat-shock proteins (Hsps), H^+^/K^+^ ATPase, H^+^/K^+^-adenosine triphosphatase, Human leukocyte antigens (HLA), chemokine receptors, Lewis antigens, and act as autoantibodies [58,67,68,69,70]. In this regard, an example could be autoimmune gastritis, in which similarities of H^+^, K^+^-ATPase and bacterial epitopes may result in the production of antibodies against parietal cells, thereby suggesting that bacterial infection may be the trigger of an autoimmune mucosal inflammation resulting to mucosa atrophy in patients with chronic *H. pylori* gastritis [71]. Moreover, Lewis antigens seem to be upregulated in the conjunctival and corneal epithelia of glaucomatous eyes [72]; chemokine receptors are overexpressed in the conjunctival epithelium of patients with glaucoma [73]; Hsps are present in retinal fractions of glaucomatous eyes [74,75]; and the expression of HLA antigens on conjunctival epithelial cells are also present in glaucomatous eyes [76,77].

The levels of the induced specific autoantibodies increase in the circulation of glaucoma patients. They may have access to the brain, due to disruption of the blood-brain barrier/blood-ocular barrier (BOB) and would then be capable of killing retinal cells. This might contribute to the development and/or progression of glaucoma [33,34,63,64,78]. Related data indicate that *H. pylori*-specific antibody levels are significantly increased in both serum and in the aqueous humor of subjects with POAG and PEG [34]. Moreover, the level of titers of anti-*H. pylori* antibody might reflect the severity of glaucomatous damage; the titer of anti-*H. pylori* IgG antibody is correlated with the degree of vertical cupping, thus possibly reflecting the severity of glaucomatous damage [34,79,80]. Due to the observed strong correlation between aqueous and serum IgG levels in POAG and PEG, it has been hypothesized that *H. pylori* antibodies circulating in the bloodstream may enter aqueous circulation due to BOB disruption and may reach levels that are possibly sufficient to impact the development and progression of glaucoma. It should be remembered that the cut-off value to determine the *H. pylori*-positive cases in aqueous humor is 4.76 U/mL [34].

In view of the aforementioned considerations, further relatively large-scale prospective studies are warranted to elucidate in depth the hypothetical role of anti-*H. pylori* antibody titer in the pathophysiology of aging-related glaucoma.

In the subgroup analysis of the present study (Table 2), the association between *H. pylori* infection and glaucoma remained significant for Eastern and Western populations. Nevertheless, heterogeneity was preserved, thus possibly reflecting *H. pylori* and host genetic polymorphisms. Relative studies in specific populations have revealed that polymorphisms of various molecules implicated in *H. pylori* infection were associated with the severity and progress of the inflammation. Genetic variations of interleukin-1β, tumor necrosis factor-α and other cytokines, normally upregulated in *H. pylori* gastritis with a pro-inflammatory and anti-secretory role, have been associated with a potentially increased risk for peptic ulcer disease and gastric cancer development in European populations. Similarly, genetic sequences of immune cells’ superficial and intracellular receptors have been incriminated for intense gastric inflammation, dysplasia and oncogenesis; rs11535889 polymorphism of Toll-like receptor-4 and rs718226 polymorphism of nucleotide-binding oligomerisation domain-like receptors (NOD)-2 have been found in different Chinese sub-populations thus predisposing to gastric cancer, whereas the E266K single nucleotide polymorphism of NOD1 was connected with intense inflammation in Korean patients [81]. Concerning the *H. pylori* virulence, two strains have been recognized with a geographical distribution. The Asian-type Cag-A strain seems to be more virulent than the respective Western-type *H. pylori*, thus possibly causing a more intense gastritis and increasing the risk of gastric cancer. Although in all Western countries the Western-type Cag-A strain predominates, in Asia there is a prompt heterogeneity. Strains of Middle East regions, India and Mongolia have the western CagA; China, Japan, Korea and Vietnam have the Asian CagA strain, whereas some regions, such as Thailand, have both Asian and western CagA strains [82,83]. As regards the glaucoma subtype, the association remained significant between *H. pylori* infection and POAG or NTG, but not PEG.

Another meta-analysis included 10 studies with 695 glaucoma patients and 1580 controls and reported similar results (OR: 2.08, *I*^2^ = 63.6%), although without clearly focusing on the potential impact of active *H. pylori* infection on glaucoma by estimation of virtual heterogeneity as well [18]. The studies added in the last five years (*n* = 5) did not change the positive association between *H. pylori* infection and glaucoma, and reinforce this relationship. It is striking that the association also remained robust for POAG or NTG, but not for PEG [18]. Future mechanistic research may shed light on this ambiguous association. Furthermore, other parameters with an established relationship to glaucoma (e.g., medications, family history, myopia, comorbidities, host genetic polymorphisms and *H. pylori* genotypes) [84] may be considered in future observational studies, to conduct multivariate analyses investigating *H. pylori* infection as a confounder of glaucoma.

From a pathogenetic point of view, potential links between *H. pylori* infection and glaucoma may include dysbiosis of the gastrointestinal microbiota and release of inflammatory cytokines, as well as chronic *H. pylori* infection and gastric inflammation that might trigger inducible release of nitric oxide synthase, nitric oxide (NO) production and formation of reactive nitrogen species, such as peroxynitrite [85]. The structural characteristics of these molecules may allow systematic diffusion and remote effects on the eye; high concentrations in the eye could promote nitrosative stress, mitochondrial damage through parthanatos (a DNA fragmenting chain reaction), neurotoxicity, optic nerve degeneration and apoptosis of retinal ganglionic cells. Furthermore, fluctuations in the severity of *H. pylori*-related gastritis may affect NO concentrations, thereby possibly modifying its vasoactive activity and contributing to unstable perfusion pressure in ocular arteries, transient ischemia and reperfusion damage, leading to increased intraocular pressure [85]. However, these speculations would have to be clarified by future research.

Finally, regarding the potential impact of *H. pylori* eradication on glaucoma parameters, a relative study reported that elimination of *H. pylori* could have effectively improve glaucoma parameters (*p* < 0.001 for intraocular pressure; *p* ≤ 0.01 for visual field), thereby suggesting a possible casual relation between *H. pylori* infection and glaucoma [42]. Likewise, comparable results were recently reported after *H. pylori* eradication by others (*p* < 0.005 for intraocular pressure) [86] Since another study reported that the effect of *H. pylori* eradication in glaucoma therapy is not clear [87], therefore further large-scale prospective studies are needed to elucidate this issue.

The current meta-analysis has some limitations. The studies included were observational, so no causal relationship could be established. Moreover, methodological differences between the studies (e.g., *H. pylori* infection diagnosis, controls selection) add to the heterogeneity, thus affecting the synthesis of the studies included. Quality assessment commonly uses NOS, but its implementation is largely subjective. Furthermore, conference abstracts and other sources of grey literature were not considered for this systematic review. On the one hand, this increases the possibility of publication bias. Moreover, the quality of data in abstracts and grey literature is not peer reviewed in-depth; as they are included, the risk of low-quality data is increased. By selecting only English publications, a selection bias is also introduced. To avoid subjective bias, a sensitivity analysis was performed after excluding all studies of our group, and the results were essentially similar to the main analysis. Another methodological concern was the controls used in some studies, who were patients with cataract or anemia. It is known that *H. pylori* infection predisposes to anemia, and its eradication is accompanied by reversal of anemia [42]. It is possible, therefore, that these control groups had higher rates of *H. pylori* infection than the general population; however, the association between *H. pylori* infection and glaucoma remained robust in studies with anemic controls.

## 5. Conclusions

In conclusion, to our knowledge, this systematic review and meta-analysis shows, for the first time, a positive association between active *H. pylori* infection and glaucoma with null heterogeneity, which is also quantified by anti-*H. pylori* titers. As a novelty prevalence, these titers rise significantly with age, and are predominantly higher among individuals aged 65 years or older [88]. This meta-analysis warrants mechanistic studies to enlighten the possible links between the two disorders, and, importantly, clinical trials to investigate the possible effect of *H. pylori* eradication on glaucoma pathophysiology. Nevertheless, since glaucoma is a multifactorial disease, we do not expect the eradication of a single factor, *H. pylori* infection, will eliminate glaucoma, but perhaps just decrease its incidence.

## Figures and Tables

**Table 1:** The characteristics of the included studies.

No	First Author	Year of Publication	Type of Study	Country (Region)	Host Journal	Glaucoma Cases	Controls	Mean Age (years) *	Controls Deposit	Glaucoma Subtype	*H. pylori* Infection Diagnosis	MD of Anti-*H. pylori* Titers	NOS Score	Relationship between *H. pylori* and Glaucoma (OR, CI)
1	Abrishami et al.	2007	Cohort	Iran (East)	*Bina J Ophthalmol*	44	79	60.8	Cataract	POAG	ELISA	N/A	N/A	3.69 (1.68–8.13)
2	Deshpande et al.	2008	Case-control	India (East)	*J Glaucoma*	50	50	63.7	Cataract	POAG	ELISA	20′220	8	1.20 (0.52–2.79)
3	Deshpande et al.	2008	Case-control	India (East)	*J Glaucoma*	50	50	67	Cataract	PEG	ELISA	−8′610		0.41 (0.18–0.91)
4	Galloway et al.	2003	Cohort	Canada (West)	*Ophthalmology*	38	94	63.2	Healthy	POAG	ELISA	−10′600	8	1.41 (0.59–3.4)
5	Galloway et al.	2003	Cohort	Canada (West)	*Ophthalmology*	16	94	73.2	Healthy	PEG	ELISA	18′500		1.32 (0.38–4.54)
6	Galloway et al.	2003	Cohort	Canada (West)	*Ophthalmology*	19	94	67.7	Healthy	NTG	ELISA	23′300		1.41 (0.45–4.4)
7	Hong et al.	2007	Case-control	China (East)	*Asian J Ophtholmol*	24	24	63.9	Healthy	POAG	UBT	N/A	8	4.49 (1.26–16)
8	Kim et al.	2011	Retrospective Cohort	South Korea (East)	*IOVS*	100	88	55.6	Healthy	NTG	ELISA	N/A	8	2.05 (1.12–3.75)
9	Kim et al.	2011	Retrospective Cohort	South Korea (East)	*IOVS*	104	1116	53.4	Healthy	NTG	ELISA	N/A		1.83 (1.17–2.86)
10	Kountouras et al.	2003	Cohort	Greece (West)	*Graefe’s Arch Clin Exp Ophthalmol*	27	31	70.6	Cataract	POAG	ELISA	37′210	8	2.35 (0.63–8.76)
11	Kountouras et al.	2003	Cohort	Greece (West)	*Graefe’s Arch Clin Exp Ophthalmol*	19	31	69.2	Cataract	PEG	ELISA	25′800		25 (1.38–452.34)
12	Kountouras et al.	2002	Cohort	Greece (West)	*Arch Intern Med*	41	30	61.4	Anemia	POAG	HISTOLOGY	18′570	8	8.23 (2.53–26.75)
13	Kountouras et al.	2001	Case-control	Greece (West)	*Ophthalmology*	32	30	64	Anemia	POAG	HISTOLOGY	N/A		8 (2.25–28.48)
14	Kountouras et al.	2001	Case-control	Greece (West)	*Ophthalmology*	9	30	62	Anemia	PEG	HISTOLOGY	N/A	7	9.14 (1.01–82.44)
15	Kurtz et al.	2008	Cohort	Israel (West)	*J Glaucoma*	13	36	67.7	Cataract	POAG	ELISA	N/A	8	0.74 (0.21–2.67)
16	Kurtz et al.	2008	Cohort	Israel (West)	*J Glaucoma*	23	36		Cataract	PEG	ELISA	N/A		1.19 (0.4–3.54)
17	Kurtz et al.	2008	Cohort	Israel (West)	*J Glaucoma*	15	36		Cataract	NTG	ELISA	N/A		0.96 (0.28–3.27)
18	Noche et al.	2016	Case-control	Cameroon (Africa)	*Ophthalmology and Eye Diseases*	50	31	58.52	Healthy	POAG	ELISA	N/A	8	0.42 (0.12–1.43)
19	Samarai et al.	2014	Case-control	Iran (East)	*Global Journal of Health Science*	37	42	73.05	Anemia	POAG	ELISA	N/A	8	5.61 (1.68–18.75)
20	Sultana et al.	2019	Case-control	Bangladesh (East)	*BSMMU J*	40	40	51.4	Healthy	POAG	ELISA	N/A	8	3.89 (1.53–9.87)
21	Tsolaki et al.	2015	Cohort	Greece (West)	*BMC Ophthalmology*	35	31	62.18	Healthy	POAG	HISTOLOGY	N/A	7	2.65 (0.97–7.24)
22	Tuzcu et al.	2015	Case-control	Turkey (East)	*Arq Bras Oftalmol*	35	35	59.08	Healthy	POAG	UBT	N/A	8	1.41 (0.55–3.62)
23	Zavos et al.	2012	Case-control	Greece (West)	*Ophthalmic Res*	51	35	71.4	Anemia	POAG	HISTOLOGY	N/A	8	5.69 (2.08–15.54)

* refers to glaucoma group, CI; Confidence Interval, ELISA; Enzyme Linked Immunosorbent Assay, *H. pylori*; *Helicobacter pylori*, MD; Differences of Means, N/A; not available, NOS; Newcastle-Ottawa scale, NTG; Normal Tension Glaucoma, OAG; Open Angle Glaucoma. OR; Odds Ratio, PEG; Pseudo-Exfoliation Glaucoma,UBT; urea breath test.

**Table 2:** Subgroup analysis regarding the relationship between *H. pylori* infection and glaucoma.

		Random Effects Model			Heterogeneity			
Subgroup	Datasets	OR	CI 95%	*p*	*Q*	*I*^2^ (%)	*t* ^2^	*p*
Total	23	2.08	1.48–2.93	*<0.001*	57.21	61.54	0.39	*<0.001*
Region								
EAST	8	2.1	1.23–3.56	*0.006*	25.1	72.11		*<0.001*
WEST	14	2.32	1.47–3.64	*<0.001*	25.51	49.04		*0.029*
AFRICA	1	0.42	0.12–1.44	*N/A*	*N/A*	*N/A*		*N/A*
Glaucoma Subtype								
POAG	14	2.57	1.66–3.99	*<0.001*	31.59	58.85		*0.003*
PEG	5	1.69	0.53–5.34	*0.373*	13.68	70.76		*0.008*
NTG	4	1.76	1.27–2.46	*<0.001*	1.36	0		*0.715*
Study Design								
Cohort	13	1.98	1.44–2.74	*0.141*	17.24	30.4		*0.141*
Case-control	10	2.33	1.13–4.81	*<0.001*	39.95	77.47		*<0.001*
Control groups								
Cataract	8	1.37	0.7–2.67	*0.362*	20.52	65.89		*0.005*
Anemia	5	6.78	3.88–11.83	*<0.001*	0.45	0		*0.98*
Healthy	10	1.82	1.33–2.5	*<0.001*	11.7	23.07		*0.231*
*H. pylori* infection diagnosis								
ELISA	16	1.57	1.09–2.28	*0.017*	35.35	57.57		*0.002*
UBT	2	2.32	0.76–7.13	*0.142*	2.06	51.35		*0.152*
HISTOLOGY	5	5.4	3.17–9.2	*<0.001*	3.02	0		*0.555*

CI; Condifence Interval, elisa Enzyme Linked Immunosorbent Assay, *H. pylori*; *Helicobacter pylori*, N/A; not available, NTG; Normal Tension Glaucoma, OAG; Open Angle Glaucoma, OR; Odds Ratio, PEG; Pseudo-Exfoliation Glaucoma, UBT; Urea Breath Test.

## Abbreviations

AD	Alzheimer’s Disease
BOB	Blood-Ocular-Barrier
CI	Confidence Interval
Hsps	heat shock proteins
H. pylori	Helicobacter pylori
HLA	Human leukocyte antigens
IG	Immunoglobulin
NOD	Nucleotide-Binding Oligomerisation Domain
NTG	Normal Tension Glaucoma
POAG	Primary Open-Angle Glaucoma
PEG	Pseudo-Exfoliation Glaucoma
RUT	Rapid Urease Test
SD	Standard Deviation
WMD	Weighted Mean Differences
